# Clinical outcomes following pencil beam scanning proton therapy for skull base chordoma and chondrosarcoma: a single-institution experience

**DOI:** 10.2340/1651-226X.2026.45297

**Published:** 2026-07-31

**Authors:** Skóra Tomasz, Kamila Rawojć, Anioł Joanna, Banaś Tomasz, Cepiga Anna, Chrostowska Agnieszka, Chudyba Adrianna, Garbacz Magdalena, Góra Eleonora, Kabat Damian, Krzywonos Emilia, Mikołajski Tomasz, Nowak-Sadzikowska Jadwiga, Patla Anna, Pluta Elżbieta, Ruciński Antoni, Urbanek Konrad, Wojton-Dziewońska Dominika, Wszołek Mariusz, Kopeć Renata, Kisielewicz Kamil

**Affiliations:** a Maria Skłodowska – Curie National Research Institute of Oncology, Kraków Branch, Kraków, Poland; b Cyclotron Centre Bronowice, The Henryk Niewodniczański Institute of Nuclear Physics, Polish Academy of Sciences, Kraków, Poland; c Faculty of Medical Sciences in Zabrze, Medical University of Silesia, Katowice, Poland

**Keywords:** skull base tumors, chordoma, chondrosarcoma, proton therapy, pencil beam scanning, radiotherapy outcomes, local control, artificial intelligence in radiotherapy, augmented reality, radiation toxicity, precision oncology, translational research, dose escalation, hypofractionation

## Abstract

**Background:**

Chordomas and chondrosarcomas of the skull base are rare, locally aggressive tumors that pose significant therapeutic challenges due to their proximity to critical anatomical structures. While maximal safe surgical resection remains the cornerstone of management, the infiltrative nature of these tumors often precludes radical excision, necessitating adjuvant radiotherapy. Given their relative radioresistance, high-dose radiotherapy exceeding 70 Gray Relative Biological Effectiveness (GyRBE) is required to achieve durable local control. This study evaluated long-term outcomes and toxicity following pencil beam scanning (PBS) proton therapy in patients with skull base chordoma and chondrosarcoma.

**Methods:**

We retrospectively analyzed 76 patients (50 chordoma, 26 chondrosarcoma) treated with curative-intent PBS between 2016 and 2020. Median prescribed doses were 74.0 GyRBE for chordomas and 70.0 GyRBE for chondrosarcomas. Clinical endpoints included overall survival (OS), local failure-free survival (LFFS), distant metastasis-free survival (DMFS), and toxicity graded according to CTCAE v5.0.

**Results:**

At a median follow-up of 70.2 months, 5-year OS, LFFS, and DMFS for the entire cohort were 84.0, 86.9, and 92.7%, respectively. Local control was numerically higher in chondrosarcoma (96.1%) than in chordoma (81.9%; *p* = 0.116). Tumor contact with the optic apparatus or brainstem was associated with worse OS (*p* = 0.0034 and *p* = 0.0151, respectively) and proximity to the optic pathways correlated with poorer local control (*p* = 0.0182). Grade ≥ 3 late toxicity occurred in 9.2% of patients, predominantly hearing impairment and temporal lobe necrosis.

**Conclusions:**

PBS achieves excellent local control and survival with an acceptable safety profile in skull base chordoma and chondrosarcoma. Our findings align with ongoing efforts toward dose intensification, hypofractionation, and particle selection. Moreover, emerging integration of artificial intelligence for contouring, treatment adaptation, and toxicity prediction, along with augmented reality-assisted positioning and patient education, promises to further enhance treatment precision and outcomes. Through long-term follow-up and engagement with international research initiatives, our institution contributes to the global advancement of biologically guided, high-precision radiotherapy for rare skull base malignancies.

## Introduction

Chordomas and chondrosarcomas are rare primary bone malignancies, accounting for approximately 6% of tumors located at the skull base [1, 2]. Chordomas originate from remnants of the notochord, which serves as the embryonic precursor to the axial skeleton, whereas chondrosarcomas arise from primitive mesenchymal cells within the cartilaginous matrix of the skull base.

Despite their distinct histogenetic origins, both tumor entities share numerous clinical, radiological, and therapeutic characteristics. Due to their expansile growth pattern, these tumors frequently infiltrate adjacent vascular and neural structures, thereby significantly limiting the feasibility of achieving complete surgical resection.

To mitigate the high risk of local recurrence, surgical intervention is commonly complemented by radiotherapy. Given the intrinsic radioresistance of both tumor types, therapeutic doses exceeding 70 Gy are generally required [3]. Achieving satisfactory local control is particularly challenging due to the proximity of radiosensitive structures such as the brainstem and optic pathways, which impose stringent dose constraints. Efforts to spare these critical organs often result in suboptimal target coverage, thereby increasing the likelihood of local failure [4].

Proton beam therapy provides a substantial solution to this therapeutic challenge. The physical characteristics of protons, including finite range, lower integral dose, and absence of exit dose, combined with the advanced delivery precision afforded by pencil beam scanning (PBS) proton therapy, enable highly conformal dose distributions. This facilitates effective tumor irradiation while sparing adjacent healthy tissues.

This study aims to report our preliminary clinical experience with PBS in patients diagnosed with skull base chordomas and chondrosarcomas.

## Materials and methods

Between November 2016 and December 2020, a total of 77 patients with skull base tumors underwent radiotherapy at the Maria Sklodowska-Curie National Research Institute of Oncology in Krakow. One patient was lost to follow-up.

The final analysis included 76 consecutive adult patients (≥18 years) with histologically confirmed chordoma (*n* = 50; 65.8%) or chondrosarcoma (*n* = 26; 34.2%). Eligibility was determined solely by the feasibility of delivering radical proton therapy with curative intent. None of the patients had received prior radiotherapy or had a history of another malignancy at the time of treatment qualification. In eight cases, the neoplastic infiltration extended to the upper cervical spine. At the time of proton therapy, 17 patients (22.4%) presented with locally recurrent disease following prior surgical intervention. The male-to-female ratio was 0.73. The median age of the study cohort was 51 years (range, 22–77), with comparable age distribution between the two histological subgroups. The majority of patients had good performance status; only six individuals (7.9%) exhibited moderate limitations in daily functioning. The most common presenting symptoms were visual disturbances (37.5%) and headache (26.6%).

Detailed demographic, clinical, and treatment-related characteristics are summarized in Table 1.

**Table 1:** Patient, tumor, and treatment characteristics.

Characteristic	All (*n* = 76)	Chordoma (*n* = 50)	Chondrosarcoma (*n* = 26)
Age, years			
Median (range)	51.0 (22–77)	51.5 (25–77)	49.5 (22–74)
Mean ± SD	50.0 ± 15.2	50.9 ± 14.4	48.3 ± 16.8
Gender			
Female	44	26	18
Male	32	24	8
Performance status (WHO)			
0	7	5	2
1	63	40	23
2	6	5	1
Optic structure involvement			
Yes	23	14	9
No	53	36	17
Brainstem involvement			
Yes	21	16	5
No	55	34	21
Tumor status			
Primary	59	37	22
Recurrent	17	13	4
GTV (ml)			
Mean ± SD	17.3 ± 24.3	14.3 ± 20.5	22.9 ± 29.9
Median (range)	10.65 (0–126.5)	8.75 (0–108.9)	12.6 (0–126.5)
Type of surgery			
Complete resection	12	10	2
Subtotal resection	62	40	22
Biopsy only	2	0	2
Presence of stabilization hardware			
Yes	4	4	0
No	72	46	26

GTV: Gross Tumor Volume.

All patients except two underwent surgical intervention prior to radiotherapy. Gross total resection was achieved in only 12 patients (15.8%). The median interval between surgical resection and initiation of proton therapy was 3.6 months (range, 0.8–21.4 months). All patients received proton radiotherapy delivered with PBS technique at the Bronowice Cyclotron Centre of the Institute of Nuclear Physics, Polish Academy of Sciences, with curative intent.

Gross Tumor Volume (GTV) was delineated based on all visible tumor remnants on diagnostic Magnetic Resonance Imaging (MRI) and planning CT. The high-risk clinical target volume (CTV-HR) encompassed residual disease and the preoperative tumor bed, along with regions at high risk for microscopic involvement. The intermediate-risk clinical target volume (CTV-IR) included the CTV-HR and adjacent areas potentially at risk for subclinical spread.

A planning target volume (PTV) margin of 3 mm was uniformly applied. Treatment Planning is performed using the Eclipse treatment planning system by Varian. For planning skull base tumors, we use the PCS (Proton Convolution Superposition) optimizer, which does not support robust planning. Therefore, uncertainty factors must be incorporated into the planning framework. It is necessary to define a PTV that compensates for all assumed uncertainties: random, systematic, and proton range uncertainty.

The margin from CTV to PTV was determined based on beam-specific range uncertainty, factoring in 3.5% of the calibration curve uncertainty, plus an additional 1 mm.

For the beam geometry used in skull base treatment plans (70°, 110°, 250°, and 290°), the calculated uncertainties were compensated using a uniform planning margin. The validity of the concept was confirmed by robust analysis.

The median total prescribed dose to the CTV-HR was 74.0 Gray Relative Biological Effectiveness (GyRBE) (range, 62.0–76.0 GyRBE) for chordomas and 70.0 GyRBE (range, 70.0–74.0 GyRBE) for chondrosarcomas. In all cases, a total dose of 54 GyRBE was prescribed to the CTV-IR. The median fractional dose was 2.0 GyRBE (range, 1.8–2.0 GyRBE).

The vast majority of patients received the intended median prescription dose. Dose escalation beyond the median was feasible in six selected cases when organ-at-risk constraints permitted; however, given the frequent proximity of tumors to critical skull base structures, this was generally limited to avoid excessive toxicity. The minimum dose of 62 GyRBE was delivered in a single patient who discontinued treatment prematurely at their own request, without objective clinical indications such as significant radiation-related toxicity.

Titanium orthopedic stabilization hardware was present within the irradiated volume in four patients.

Initially, a preliminary study was performed on a phantom containing a metal stabilizer to determine which image artifact reduction system is best. GE MARS (dual-energy) and Siemens’ iterative metal artifact reduction (iMAR) algorithms were compared, with iMAR providing superior artifact suppression. Following the development of a dedicated calibration curve for the treatment planning system, iMAR was implemented in clinical proton therapy planning.

For each beam, a dedicated field-specific target structure was generated to avoid beam traversal through metallic components whenever feasible. All plans underwent dosimetric verification. In patients with stabilization hardware, additional Monte Carlo dose calculations using the Fast paRticle thErapy Dose evaluator (FRED) code confirmed satisfactory agreement.

MRI scan was used for anatomical structures delineation and was not suppressed for artifacts in any way.

Following treatment, all patients were placed under routine surveillance at our institution. Follow-up included clinical evaluation and brain MRI every 3–6 months to assess local control, and thoracic and abdominal CT scans at least annually to exclude distant dissemination.

Toxicity was assessed according to CTCAE version 5.0. Late radiation toxicity was defined as adverse events (AEs) occurring more than 90 days after completion of proton therapy.

Treatment efficacy was assessed based on the following outcomes: overall survival (OS), disease-free survival (DFS), local failure-free survival (LFFS), and distant metastasis-free survival (DMFS). Survival times were calculated from the first day of radiotherapy until death from any cause, or the first occurrence of local or distant failure. Local failure was defined as radiologically confirmed tumor progression or recurrence at the primary site, characterized by an increase in lesion size and/or the appearance of new contrast-enhancing tumor tissue within or adjacent to the irradiated volume on follow-up MRI. Five-year survival rates were estimated using the Kaplan-Meier method. The log-rank test (Peto method) was used to assess statistical significance, with a *p*-value of <0.05 considered significant. Statistical analyses were conducted using Statistica v13 (TIBCO Software Inc., 2017).

## Results

The median follow-up, calculated from the initiation of radiotherapy, was 70.2 months (range: 2.8–94.6 months). At the time of analysis, 58 patients (76.3%) were alive, of whom five presented with active disease. The shortest follow-up of 2.8 months corresponded to a patient who died from a non-neoplastic cause shortly after completion of radiotherapy. Of the 18 recorded deaths, 9 were related to tumor progression, whereas 9 resulted from non-oncologic causes.

**Figure 1:**
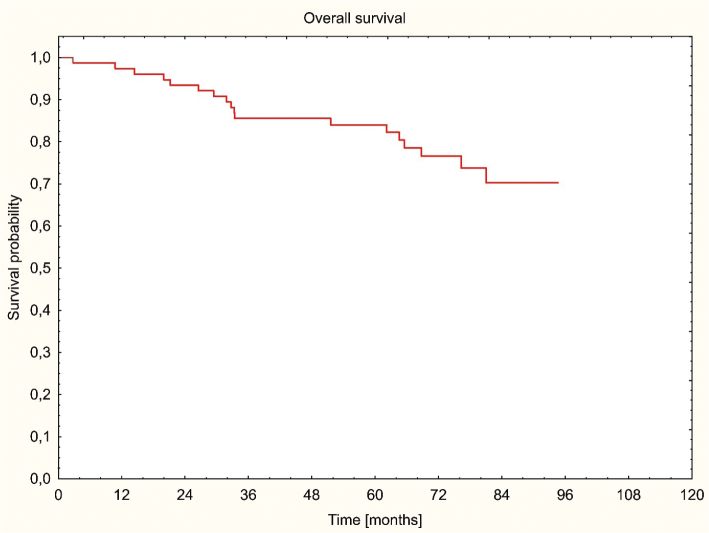
Kaplan–Meier curves for Overall Survival (OS) in chondrosarcomas and chordomas.

The estimated 3- and 5-year OS rates for the entire cohort were 85.5 and 84.0%, respectively (Figure 1). In the chordoma subgroup, the corresponding values were 84.0 and 81.7%, whereas in patients with chondrosarcoma, both the 3- and 5-year OS rates were 88.5%. The difference in OS between histological subgroups was not statistically significant (log-rank *p* = 0.41).

**Figure 2:**
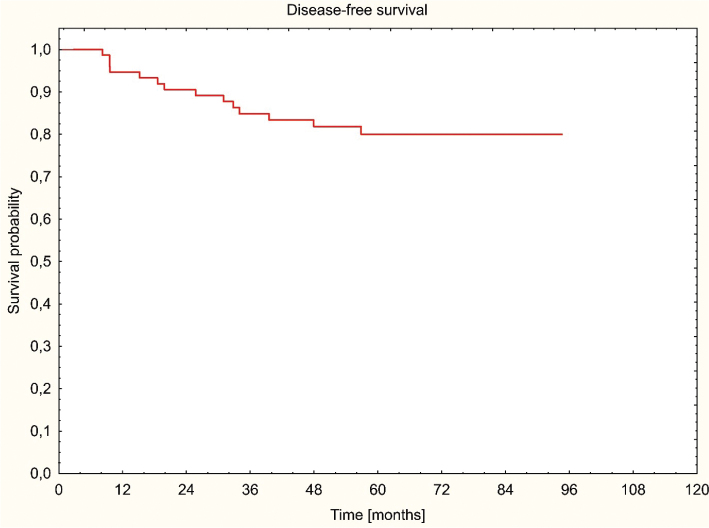
Kaplan-Meier curves for Disease-Free Survival (DFS) in cohort.

The 5-year disease-free survival (DFS) rate for the entire cohort was 80%. DFS was 25% higher among patients with chondrosarcoma compared to those with chordoma. This difference was statistically significant (71.3 vs. 96.1%*p* = 0.017) (Figure 2).

**Figure 3:**
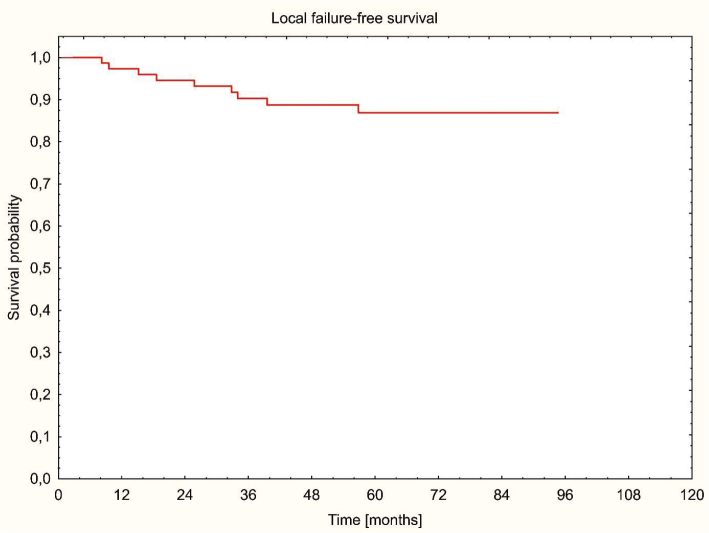
Kaplan-Meier curves for Local Failure-Free Survival (LFFS).

The estimated 3- and 5-year LFFS rates were 90.2 and 86.9%, respectively (Figure 3). The median time to local failure was 25.7 months, with chordomas showing a median of 29.3 months and chondrosarcomas 9.5 months. Eight local failures were observed in patients with chordoma, while only one local recurrence occurred in the chondrosarcoma subgroup.

In seven of the chordoma cases and in the single chondrosarcoma case, recurrence occurred within the high-risk target volume. In one chordoma patient, recurrence occurred outside the initial GTV, within the postoperative surgical bed that had been included in the CTV-IR.

The 5-year LFFS rate among chordoma patients was approximately 15% lower than in those with chondrosarcoma (81.9 vs. 96.1%), though this difference did not reach statistical significance (*p* = 0.116).

**Figure 4:**
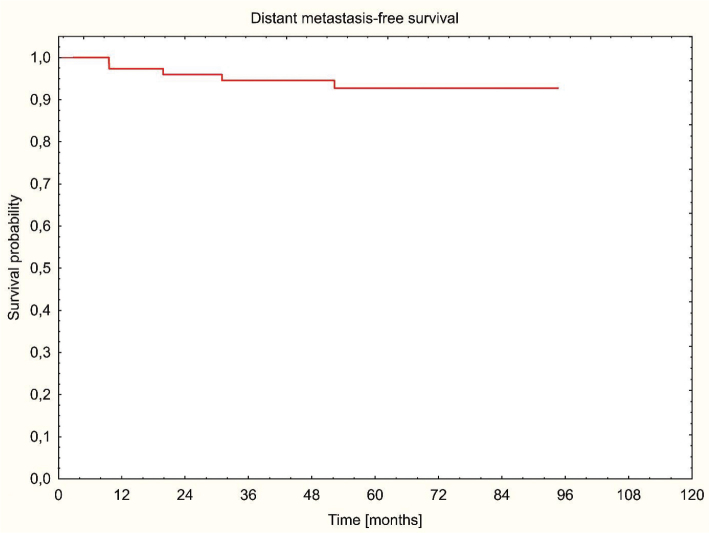
Kaplan-Meier curves for Distant Metastasis-Free Survival (DMFS).

Distant metastases were observed in five patients (6.6%), all of whom had chordoma. In three cases, metastases were located in bone, while the remaining two presented with non-osseous dissemination – one to the soft tissues of the neck, and the other with extraspinal metastasis within the spinal canal. The median time to distant metastasis was 19.9 months. The 3- and 5-year DMFS rates were 94.5 and 92.7%, respectively (Figure 4).

Multivariate analysis confirmed the prognostic significance of tumor proximity to critical anatomical structures. Direct contact of the tumor with either the optic apparatus or the brainstem was associated with significantly worse OS, with hazard ratios (HR) of 5.33 (95% CI: 1.74–16.29; *p* = 0.0034) and 3.39 (95% CI: 1.27–9.05; *p* = 0.0151), respectively. Furthermore, tumor proximity to the optic pathways was associated with a nearly fivefold increase in the risk of local recurrence (HR = 4.88; 95% CI: 1.31–18.22; *p* = 0.0182).

**Figure 5:**
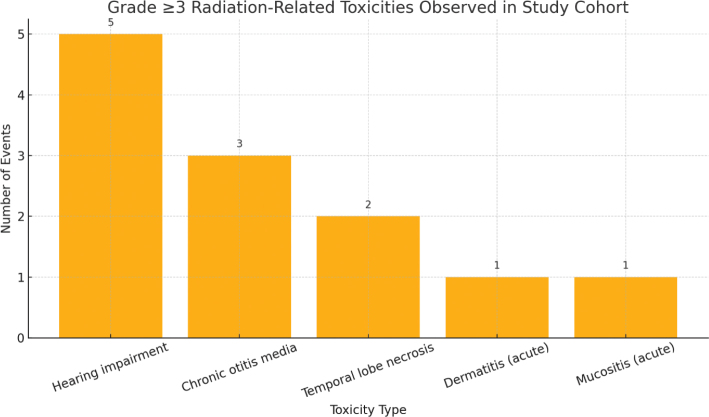
Radiation-Related Toxicities in the study cohort.

Treatment was generally well tolerated. No patient required treatment interruption due to acute radiation toxicity. Acute toxicity was predominantly mild to moderate, with two cases of grade 3 events. Late radiation toxicity was observed in 63 patients (82.9%) over the course of extended follow-up, including seven grade 3 complications (Table 2). No grade 4 or 5 late toxicities were reported. The median time to onset of severe late toxicity was 60.3 months (range: 17.4–79.5 months). The 5-year rate of freedom from grade ≥ 3 late toxicity was 93.8%, with no significant differences between patients with chordomas and those with chondrosarcomas (Figure 5).

**Table 2:** Radiation-related grade 3 toxicity (CTCAE v5.0).

Type of Toxicity	Number of Events
Acute	
Dermatitis	1
Mucositis	1
Late	
Hearing impairment (unilateral)	5
Chronic otitis media	3
Temporal lobe necrosis	2

Among grade ≥ 3 treatment-related toxicities, unilateral hearing impairment was the most frequent AE, followed by chronic otitis media and temporal lobe necrosis, all occurring at low frequencies, underscoring the acceptable safety profile of high-dose proton therapy in this cohort.

In addition to grade ≥ 3 toxicities, our cohort demonstrated a substantial incidence of grade 1–2 AEs, which are clinically relevant for long-term quality of life. The most common low-grade events included dermatitis (23.7%), mucositis (17.1%), xerostomia (10.5%), and mild cognitive or memory complaints (7.9%). Radiation necrosis was observed radiologically in four patients (5.3%), two of whom were symptomatic (grade 2) and managed conservatively with corticosteroids. Importantly, no cases of necrosis within the brainstem or optic apparatus were detected, underscoring the relative safety of PBS in this anatomically constrained region.

Three grade 1 and two grade 2 cases of hypothalamic–hypophyseal axis dysfunction were documented during follow-up. Given that a proportion of patients continued surveillance outside our institution and routine hormonal axis testing is not uniformly performed in regional centers, mild or subclinical hypothalamic–hypophyseal insufficiency may be underreported.

## Discussion

Chordomas and chondrosarcomas of the skull base are located in close proximity to numerous critical anatomical structures, including the brainstem and optic apparatus. Radical surgical resection of these tumors is often associated with a high risk of severe neurological complications, rendering complete excision unfeasible in over half of the cases [5–9]. The limited potential for achieving gross total resection necessitates a multimodal treatment approach in which radiotherapy constitutes a key adjunctive component. The primary objective of such combined therapy is to improve local tumor control and thereby enhance long-term survival outcomes.

Historical data from the late 20th century indicate that adjuvant photon radiotherapy, delivered using conventional 2D or 3D techniques following resection of skull base tumors, offered limited efficacy in achieving durable local control [10, 11]. According to observations by Pearlman, optimal control in patients with chordoma required doses ≥80 Gy, whereas doses in the 40–60 Gy range yielded only a palliative effect in approximately one-third of cases [12]. Similar conclusions were drawn by Higinbotham and colleagues, who recommended therapeutic doses of at least 70 Gy based on a 35-year clinical follow-up [13].

Technological limitations at that time, coupled with the physical characteristics of photon beams, precluded safe dose escalation in regions adjacent to radiosensitive organs. The lack of conformality in dose distribution led to frequent violations of dose constraints for critical structures, resulting in unacceptable levels of treatment-related toxicity. These challenges stimulated interest in alternative radiation modalities such as proton beam therapy, which exploits the Bragg peak phenomenon to deliver high radiation doses to the target while sparing surrounding normal tissues. This advantage has been confirmed in systematic reviews, reporting 5-year local control rates approaching 80% for skull base tumors treated with proton therapy [14]. Comparable organ-at-risk sparing with proton-based strategies has also been demonstrated in chordomas outside the skull base, where reduced rates of selected late toxicities were observed compared with photon techniques [15].

The present retrospective analysis evaluates the clinical outcomes of 76 patients with chordoma or chondrosarcoma of the skull base treated with scanning beam proton therapy at the National Research Institute of Oncology in Krakow.

The demographic and clinical characteristics of our cohort are broadly consistent with previously published data. The median patient age exceeded 40 years and chordomas were more prevalent than chondrosarcomas. Most cases involved primary tumors, and gross total resection was achieved in only a minority. Approximately one-third of tumors demonstrated proximity to critical structures such as the brainstem or optic apparatus. Radiotherapy, delivered at doses of 70–74 GyRBE, was primarily administered as adjuvant treatment. Notably, our cohort had a relatively small average residual tumor volume (mean GTV 17.3 ± 24.3 ml), which may carry favorable prognostic implications [7–9, 16].

Given the locally infiltrative growth pattern of these tumors, long-term local control remains a major determinant of treatment success and overall prognosis. Our 5-year local control rates of 81.9% for chordoma and 96.1% for chondrosarcoma are consistent with results reported in the literature [7, 17–20]. In one of the largest published series from the Paul Scherrer Institute (PSI), which included 222 patients treated with scanning proton beams, the 5-year local control rates were 75.8% for chordomas and 93.6% for chondrosarcomas [8]. The observed ~15% difference in local control between the two tumor types reflects the more aggressive biological behavior of chordomas, which are associated with a higher risk of recurrence despite multimodal treatment and dose escalation.

One of the key factors limiting local control in skull base tumors treated with proton therapy is the proximity of the target volume to organs at risk. The permissible tolerance doses for many of these structures are up to 25% lower than the therapeutic doses required for effective tumor control, thereby constraining the extent of dose escalation in clinical practice. In a multivariate analysis by Weber et al., tumor abutment of the brainstem or optic pathways was associated with a fourfold increased risk of local failure (HR = 4.11; 95% CI: 1.79–9.44; *p* = 0.0009), and a significant reduction in OS (HR = 2.68; 95% CI: 1.13–6.34; *p* = 0.0244) [8]. Similar findings were reported by Hug et al., who demonstrated a decrease in 5-year local control from 94 to 53% when the tumor abutted the brainstem (*p* = 0.04) [21]. Our analysis similarly confirmed the negative prognostic impact of tumor contact with the optic pathways on both OS (*p* = 0.0034) and local control (*p* = 0.0182), as well as with the brainstem on OS (*p* = 0.0151). These findings underscore the importance of achieving maximal safe resection, particularly in the vicinity of critical structures, to facilitate subsequent dose escalation during proton therapy.

The role of definitive particle therapy as a surgery-sparing strategy in skull base chordomas and chondrosarcomas remains controversial. Definitive proton therapy may offer theoretical advantages, including preservation of native anatomy enabling more precise MRI-based GTV delineation and avoidance of surgical tissue disruption, which could potentially reduce susceptibility of surrounding structures to radiation-related complications.

However, available clinical evidence continues to support maximal safe resection whenever feasible. Several proton series have demonstrated improved outcomes in patients with smaller residual tumor volumes and greater separation from critical organs at risk [8, 20]. Surgical debulking may therefore improve the therapeutic ratio by reducing tumor burden and facilitating safe high-dose delivery in anatomically constrained skull base regions.

Carbon-ion therapy, owing to its higher linear energy transfer and increased relative biological effectiveness, represents a biologically attractive option in radioresistant tumors such as chordoma. Although retrospective data in skull base and sacral disease [16, 22–26] report promising outcomes, prospective comparative evidence remains lacking.

In our cohort, only two patients were treated without prior resection, precluding meaningful comparison. Thus, our results do not support routine surgery-sparing proton therapy but rather reinforce the importance of multidisciplinary management combining maximal safe resection with high-dose particle therapy.

Accurate assessment of late radiation-related toxicity requires prolonged follow-up. According to the literature, the incidence of grade ≥ 3 late complications ranges from 3 to 16.7% [8, 21–23, 27]. In our cohort, grade 3 toxicities were observed in seven patients (9.2%). Five patients (6.6%) developed unilateral hearing loss requiring the use of a hearing aid, while two (2.6%) were diagnosed with temporal lobe radionecrosis, resulting in moderate impairment of short-term memory and psychomotor performance. The overall rate of radiologically detected temporal lobe changes is comparable with reports from other proton centers, where the incidence of temporal lobe necrosis varies between 3 and 12% depending on dose–volume relationships [7, 21, 27]. Importantly, no cases of radiation-induced injury to the optic pathways or brainstem were documented. These findings are consistent with previous reports, which describe optic neuropathy rates generally below 5% [8, 9, 17, 26], and brainstem toxicity appears uncommon. In a large single-institution analysis of 163 patients treated with dose-escalated conformal proton therapy, Holtzman et al. reported a 5-year cumulative incidence of grade ≥ 2 brainstem toxicity of only 1.3%, despite median prescribed doses exceeding 73 GyRBE, with no irreversible events observed [28].

As with any retrospective study, our analysis has certain methodological limitations that should be considered when interpreting the results. These include the single-institution design and the heterogeneity of the cohort, particularly the inclusion of more than 20% of patients treated for recurrent disease. Additionally, long-term follow-up was conducted partially outside the primary center, potentially affecting the completeness and quality of data on late toxicity and limiting consistent follow-up verification. Recognizing these limitations, we continue to expand our patient database, which will enable future analyses of more homogeneous subgroups and provide broader insight into long-term treatment outcomes.

## Emerging clinical trials and translational directions in skull base tumor therapy

This section highlights the alignment of our institutional research with current global advancements in precision oncology and proton therapy. It contextualizes our findings within a broader translational framework and signals readiness for integration of next-generation therapeutic strategies.

Table 3 summarizes recent and ongoing clinical trials that illustrate the evolving landscape of skull base chordoma and chondrosarcoma management. Several studies focus on optimizing radiotherapy regimens – most notably a phase I/II trial conducted at Washington University and the University of Pennsylvania that validated dose-escalated proton therapy with a median dose of 73.8 GyRBE, achieving high local control with acceptable toxicity.

**Table 3:** Selected clinical trials relevant to skull base chordomas and chondrosarcomas (2020–2025).

Trial/Reference	Therapeutic strategy	Design/Phase	Population	Key objectives
University of Pennsylvania, Philadelphia, US (NCT01449149)	PBS Proton Therapy (72–79.2 GyRBE)	Phase I/II (Completed)	Skull base chordoma/chondrosarcoma	Evaluate safety and efficacy of high-dose proton therapy
Centro de Protonoterapia Quironsalud, Madrid, Spain (NCT05861245)	Ultra-hypofractionated Proton Therapy (5 fx)	Phase II (Ongoing)	Newly diagnosed chordoma/chondrosarcoma	Assess feasibility and toxicity of 5-fraction regimens
University of Heidelberg, Germany (NCT01182779)	Proton vs Carbon Ion Therapy	Prospective Comparative	Skull base chordoma	Compare local control and toxicity profiles
NIH Clinical Center, Bethesda, US (NCT01519817)	Antigen-Specific Immunotherapy (MVA-brachyury)	Phase I (Completed)	Advanced chordoma	Assess safety and immune response to brachyury vaccine
MGH, Boston, US (NCT01407198)	Nilotinib (Tyrosine Kinase Inhibitor BCR-ABL)	Phase I (Early phase)	Refractory chordoma	Evaluate repurposed TKI efficacy in high-risk tumors
European Multicenter Trial (NCT03083678)	Afatinib (EGFR-Targeted Therapy)	Phase II (completed)	Metastatic chordoma	Test response in EGFR-overexpressing chordoma
UCLA, Los Angeles, US (NCT03623854)	Nivolumab + Relatlimab (anti–PD-1 + anti–LAG3)	Phase II (Ongoing)	PD-L1–positive advanced chordoma	Assess combined checkpoint inhibition in chordoma

PBS: pencil beam scanning; MVA: Modified Vaccinia Ankara; BCR-ABL: Breakpoint Cluster Region-Abelson Tyrosine Kinase; EGFR: Epidermal Growth Factor Receptor; UCLA: University of California, Los Angeles; PD-L1: Programmed Death-Ligand 1

Currently, a prospective phase II trial (NCT05861245) is exploring ultra-hypofractionated proton therapy protocols for chordomas and chondrosarcomas, delivering treatment in just five fractions. This approach, enabled by the conformality of PBS–Proton Beam Therapy (PBT), aims to improve patient convenience while preserving efficacy and safety.

In parallel, the HIT-1 trial (NCT01182779) investigates whether carbon ion therapy – owing to its higher linear energy transfer – confers superior local control compared to protons in skull base chordomas. These trials underscore the field’s shift toward biological dose intensification while minimizing exposure to critical structures.

Systemic and immunotherapeutic strategies are also under active exploration. A completed phase I trial (NCT01519817) evaluated a brachyury-targeted cancer vaccine, exploiting the tumor-specific expression of the T gene product in chordomas. In the realm of targeted therapies, ongoing efforts are testing agents such as nilotinib – a tyrosine kinase inhibitor previously used in leukemia – for efficacy in refractory chordomas.

Epidermal Growth Factor Receptor (EGFR) inhibition has emerged as a potential systemic strategy in EGFR-positive chordomas, with agents such as cetuximab and afatinib under investigation. Furthermore, immunotherapy trials exploring checkpoint blockade (e.g. nivolumab plus anti-Lymphocyte Activation Gene 3 [LAG3] relatlimab) for Programmed Death-Ligand 1 (PD-L1)–expressing chordomas are currently underway (e.g. NCT04642945), indicating growing interest in integrating immunomodulation with high-precision local therapies.

Collectively, these studies illustrate a translational shift toward biologically informed, personalized treatment paradigms in chordoma and chondrosarcoma. The future lies in integrating local (proton/carbon therapy) and systemic (targeted, immune) strategies, with the potential to tailor therapy based on molecular markers, radiomic signatures, and clinical risk models. Emerging technologies including artificial intelligence for contouring, motion management, toxicity prediction, augmented reality-assisted visualization, and virtual reality-based treatment planning are expected to further improve precision and personalization of particle therapy [29–34].

## Conclusions

This study demonstrates that PBS proton therapy offers an effective and safe treatment strategy for patients with skull base chordomas and chondrosarcomas. With a median follow-up of over 70 months, our institution achieved high rates of local control and OS, accompanied by a relatively low incidence of late high-grade toxicity. Additionally, the proximity of the tumor to radiosensitive structures such as the brainstem and optic apparatus emerged as a major determinant of both treatment outcome and the potential for dose escalation. These findings underscore the clinical value of precision radiotherapy techniques in anatomically constrained regions such as the skull base.

To conclude, our clinical experience contributes to the ongoing research worldwide supporting the use of proton therapy in the management of skull base chordomas and chondrosarcomas. Our findings are complementary to the outcomes reported in international clinical trials exploring advanced radiotherapy techniques, including dose-escalated proton therapy, ultra-hypofractionation protocols, and comparative studies with carbon ion therapy.

## Data Availability

The data underlying this study are not publicly available due to the inclusion of sensitive patient information and applicable data protection regulations. Anonymized data may be made available from the corresponding author upon reasonable request and subject to institutional approval and compliance with relevant ethical and legal requirements.

## Future directions

As research efforts worldwide increasingly focus on integrating molecular and immunological targets – such as brachyury-directed vaccines and immune checkpoint inhibitors – our institution aims to align its clinical and translational activities with these developments. Through rigorous long-term outcome monitoring and growing involvement in collaborative registries and consortia, we seek to strengthen the collective understanding of optimal therapeutic strategies for these rare and anatomically complex tumors.

Taken together, our results further support the role of high-precision proton therapy as a key component of multidisciplinary treatment for skull base chordomas and chondrosarcomas. While durable tumor control can be achieved in a substantial proportion of patients, outcomes remain closely linked to the anatomical relationship between the tumor and adjacent critical neural structures, which continues to define the limits of safe dose escalation. In this context, ongoing improvements in surgical techniques, particle therapy delivery, and collaborative outcome reporting will be essential to further refine therapeutic strategies and improve long-term outcomes for patients with these rare and anatomically challenging malignancies.
